# A Concept Suggestion on the Effect of Cigarette Smoking in Inflammatory Destruction of Gingiva

**DOI:** 10.30699/IJP.14.2.184

**Published:** 2019-06-10

**Authors:** Noushin Jalayer Naderi

**Affiliations:** *Department of Oral and Maxillofacial Pathology, Faculty of Dentistry, Shahed University, Tehran, Iran*

**Dear Editor,**

Cigarette smoking has destructive effect on periodontal tissue. The rates of loss of periodontal attachment and recession of gingival are higher in smokers than non-smokers (1-2). Previous studies on the inflammatory immune responses in smokers’ periodontitis have mainly focused on the role of neutrophils. Tumor necrosis factor–α, prostaglandin E2 and matrix metalloproteinase-8 have been shown to rise in smokers with periodontitis (3-4). 

Different functions of mast cells and eosinophils in inflammatory immune responses make them distinctive cells in disease pathogenesis (5-6).

In an investigation, our team examined the effect of smoking on mast cells density in chronic periodontitis. The study showed that the mean number of mast cells in smokers was significantly lower compared to the non-smokers. Based on the literature, no research was found regarding the effect of cigarette smoking on eosinophil cells in human periodontitis. Eosinophils and mast cells regulate the hypersensitivity reactions by affecting each other function (5). Thus, in the next study, we examined this issue on the same samples. The results revealed that the number of eosinophil count in smokers was significantly lower than non-smokers.

Considering the findings of both studies on decreased number of mast cells and eosinophils in the same samples, it seems that cigarette smoke had an apoptotic function on extra-vascular immune inflammatory related cells in human periodontitis.

According to our opinion, with the death of mast cells and eosinophils, a cascade of different events occurs in the microenvironment of gingiva which causes more tissue damage in the smokers. The apoptotic effect of cigarette smoke on gingival connective tissue must be studied in the enzymatic level. The Heme Oxygenase-1 (HO-1)/Carbon Monoxide (CO) system demand to explain the pathogenesis of diseases by using the basic metabolism and enzymatic activities. HO-1 has a regulatory action on inflammatory signaling programs. CO is an end-product of HO-1. CO affects the apoptosis and cellular inflammation by modulating the inflammatory related cytokines. Modulating the HO-1 and application of CO-releasing molecules are new therapeutic strategies in inflammatory diseases (7).

Based on our previous findings, we suggest that further study on HO-1/CO can probably determine the effect of cigarette smoke on inflammatory immune cells in human chronic periodontitis. The system can be potentially considered as a therapeutic target in inflammatory disease of periodontium in cigarette smokers.

## Conflict of Interest

The authors declared no conflict of interest regarding the publication of this article.
